# Tryptamine, a Microbial Metabolite in Fermented Rice Bran Suppressed Lipopolysaccharide-Induced Inflammation in a Murine Macrophage Model

**DOI:** 10.3390/ijms231911209

**Published:** 2022-09-23

**Authors:** Afifah Zahra Agista, Sharon Angela Tanuseputero, Takuya Koseki, Ardiansyah, Slamet Budijanto, Halima Sultana, Yusuke Ohsaki, Chiu-Li Yeh, Suh-Ching Yang, Michio Komai, Hitoshi Shirakawa

**Affiliations:** 1 Laboratory of Nutrition, Graduate School of Agricultural Science, Tohoku University, 468-1 Aramaki Aza Aoba, Aoba-ku, Sendai 980-8572, Japan; 2 School of Nutrition and Health Sciences, Taipei Medical University, Taipei 11031, Taiwan; 3 Faculty of Agriculture, Yamagata University, Tsuruoka 997-8555, Japan; 4 Department of Food Technology, Universitas Bakrie, Jakarta 12920, Indonesia; 5 Faculty of Agricultural Engineering and Technology, IPB University, Bogor 16680, Indonesia; 6 International Education and Research Center for Food Agricultural Immunology, Graduate School of Agricultural Science, Tohoku University, 468-1 Aramaki Aza Aoba, Aoba-ku, Sendai 980-8572, Japan

**Keywords:** aryl hydrocarbon receptor, fermented rice bran, tryptamine, tryptophan

## Abstract

Fermentation is thought to alter the composition and bioavailability of bioactive compounds in rice bran. However, how this process affects the anti-inflammatory effects of rice bran and the bioactive compounds that might participate in this function is yet to be elucidated. This study aimed to isolate bioactive compounds in fermented rice bran that play a key role in its anti-inflammatory function. The fermented rice bran was fractionated using a succession of solvent and solid-phase extractions. The fermented rice bran fractions were then applied to lipopolysaccharide (LPS)-activated murine macrophages to evaluate their anti-inflammatory activity. The hot water fractions (FRBA), 50% ethanol fractions (FRBB), and *n*-hexane fractions (FRBC) were all shown to be able to suppress the pro-inflammatory cytokine expression from LPS-stimulated RAW 264.7 cells. Subsequent fractions from the hot water fraction (FRBF and FRBE) were also able to reduce the inflammatory response of these cells to LPS. Further investigation revealed that tryptamine, a bacterial metabolite of tryptophan, was abundantly present in these extracts. These results indicate that tryptamine may play an important role in the anti-inflammatory effects of fermented rice bran. Furthermore, the anti-inflammatory effects of FRBE and tryptamine may depend on the activity of the aryl hydrocarbon receptor.

## 1. Introduction

Inflammation is a condition associated with various ailments and abnormalities in the body, such as diabetes [1,2], sarcopenia [3], and inflammatory bowel disease [4]. It is a protective mechanism of body tissues against pathogen invasion and cell damage. However, an uncontrolled inflammatory response may cause unfavorable outcomes in individuals [5]. Inflammation is initiated by the innate immune system, including the immune cells that reside in tissues, such as macrophages, mast cells, fibroblasts, and dendritic cells, along with circulating leukocytes [6,7,8,9]. Macrophages play an important role in the pathogenesis of inflammation because of their polarized nature. While there is a spectrum of macrophage responses to bodily injuries and infection, macrophages can generally be categorized into two main groups, M1 and M2. The M1 macrophages are active during infection or injury. They also produce a range of pro-inflammatory cytokines, such as IL-1β, IL-6, inducible nitric oxide synthase (iNOS), and TNF-α [10,11]. Meanwhile, M2 macrophages are involved in tissue remodeling and immunoregulation [10]. Considering that inflammation plays a vital role in the body’s regulation and well-being, bioactive compounds that can modulate inflammation have been well studied.

Rice bran, a by-product of the rice milling industry, is well known for its nutritional quality. It contains a myriad of bioactive compounds with various functional properties, such as polysaccharides [12], sterols, and polyphenols [13]. Several studies have shown that rice bran has anti-inflammatory effects [14,15]. Currently, the use of rice bran for human consumption is limited. The limitation of its usage is mainly due to its instability, making it difficult to incorporate rice bran into various food products [16,17,18]. In the absence of treatment, rice bran tends to develop rancidity. This has proven to be a challenge in its development as a food ingredient and supplement [16,17,18]. In this study, a dual fermentation process was introduced to make rice bran more suitable for use as a food ingredient or supplement.

A dual fermentation with *Aspergillus kawachii* and a mixture of lactic acid bacteria was used to produce fermented rice bran (FRB). *Aspergillus kawachii* is a fungus that is commonly used in the fermentation process of shochu, a distilled spirit from Japan, and doenjang, a fermented soybean paste from Korea [19,20]. *Aspergillus kawachii* produces various hydrolytic enzymes, such as amylase, protease, and lipase, throughout the fermentation process. These enzymes degrade complex molecules found in either rice, barley, or soybean into smaller molecules. These smaller molecules are then used by other microbes, such as *Saccharomyces cerevisiae* or *Bacillus subtilis* [19,20]. In this study, the products digested by *Aspergillus kawachii* were further utilized by lactic acid bacteria. Some strains of lactic acid bacteria, such as *Lacticaseibacillus rhamnosus*, *Levilactobacillus brevis*, and *Enterococcus faecium*, are commonly known to act as probiotics and can be found in various fermented foods [21,22,23,24,25]. This combination of microbes was expected to enhance the nutritional quality of rice bran.

Previous studies have revealed that FRB is capable of protecting mouse intestines from dextran sodium sulfate-induced colitis [4] and reducing inflammation markers in the muscles of diabetic rats [3]. This particular fermentation process has been reported to enrich the nutritional quality of rice bran, specifically its total phenolic content [26]. It also enhances the levels of tryptophan and tryptophan microbial metabolites in rice bran [4]. These changes in the nutritional quality might contribute to the anti-inflammatory effects of FRB. However, the specific compounds in FRB that contribute to this protection against inflammation remain unknown. This study aimed to isolate the bioactive compounds in FRB that play a key role in its anti-inflammatory function.

## 2. Results

### 2.1. Non-Fermented Rice Bran (RB) and FRB Ameliorate Lipopolysaccharide (LPS)-Induced Pro-Inflammatory Cytokine Expression

**Figure 1:**
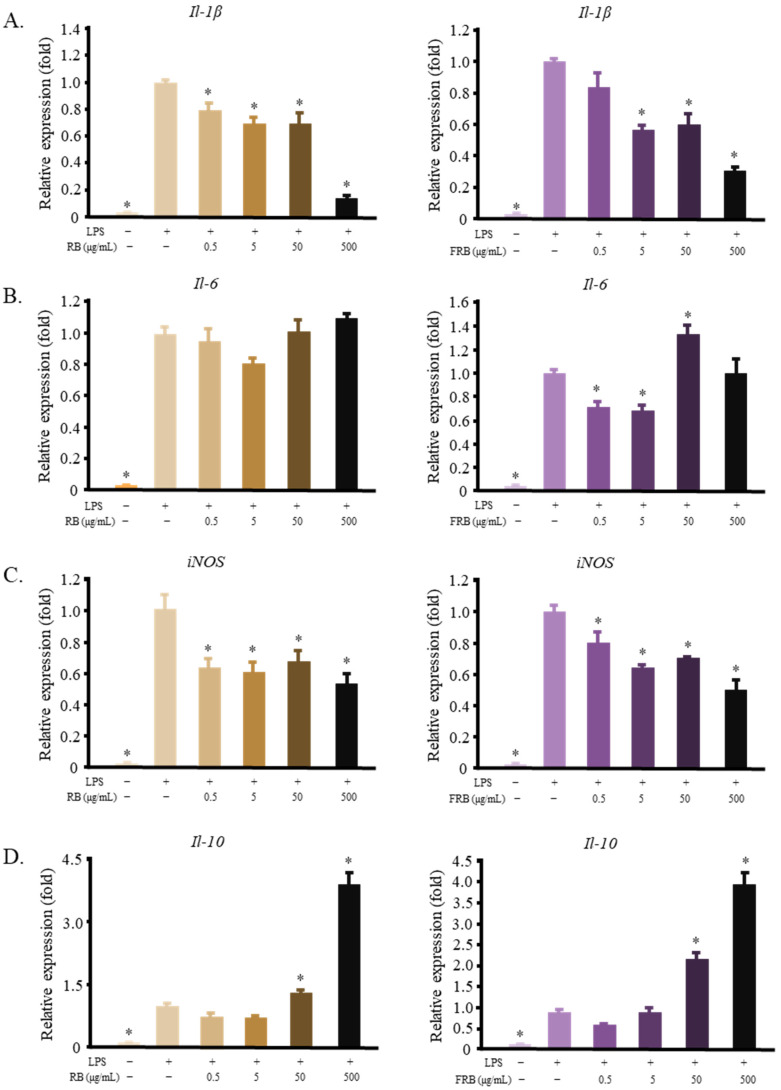
Both RB and FRB decrease the inflammatory reaction in LPS-stimulated RAW 264.7 cells. (**A**) RB and FRB lowered the mRNA level of *Il-1β*; (**B**) RB did not affect the expression of *Il-6*, while FRB reduced the mRNA level of *Il-6*; (**C**) Both RB and FRB decreased the expression of *iNos*; (**D**) RB and FRB increased the expression of *Il-10*. The data are represented as mean ± SE, *n* = 3. Significant differences between the LPS group and the other groups (Dunnet’s analysis, *p* < 0.05) are marked with asterisks (*).

The anti-inflammatory activities of both RB and FRB were assessed at this stage of the experiment. This evaluation was performed to determine whether fermentation had any effect on the anti-inflammatory activity of RB. Both RB and FRB were able to decrease the mRNA levels of *Il-1β* and *iNos* (Figure 1A,C) while simultaneously increasing the level of *Il-10* (Figure 1D). Thus, it is safe to assume that both RB and FRB possess anti-inflammatory properties. However, only FRB, but not RB, affects the level of *Il-6* (Figure 1B). This effect may be attributed to the effect of the fermentation process on modifying the bioactive compounds in rice bran.

### 2.2. Solvent-Based Fractionation of FRB

**Figure 2:**
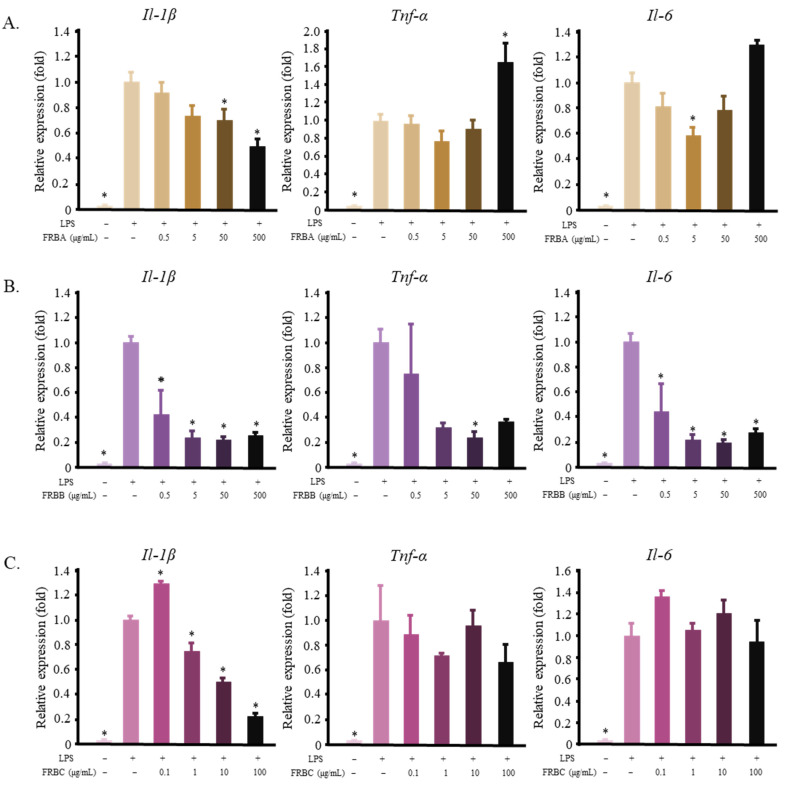
Solvent extraction of FRB results in fractions that reduce the mRNA level of pro-inflammatory cytokines in LPS-activated RAW 264.7 cells. (**A**) FRBA decreased the mRNA level of *Il-1β* and *Il-6* while increasing the level of *Tnf-α*; (**B**) FRBB reduced the expression of *Il-1β*, *Tnf-α*, and *Il-6*; (**C**) FRBC lowered the expression of *Il-1β* but did not affect *Tnf-α* or *Il-6*. The data are represented as mean ± SE, *n* = 3. Significant differences between the LPS group and the other groups (Dunnet’s analysis, *p* < 0.05) are marked with asterisks (*).

The FRBA, FRBB, and FRBC from a solvent-based fractionation were used as a pre-treatment in RAW 264.7 cells before the cells were stimulated with LPS. Changes in the mRNA expression of pro-inflammatory cytokines were measured to evaluate their anti-inflammatory activity. FRBA lowered the expression of *Il-1β* and *Il-6*; however, it also increased the level of *Tnf-α* at a concentration of 500 μg/mL (Figure 2A). In contrast, FRBB decreased the mRNA level of *Il-1β*, *Tnf-α*, and *Il-6* (Figure 2B). Meanwhile, FRBC was only able to reduce the level of *Il-1β* but did not affect the level of *Tnf-α* or *Il-6* (Figure 2).

### 2.3. Solid-Phase Fractionation of FRB

**Figure 3:**
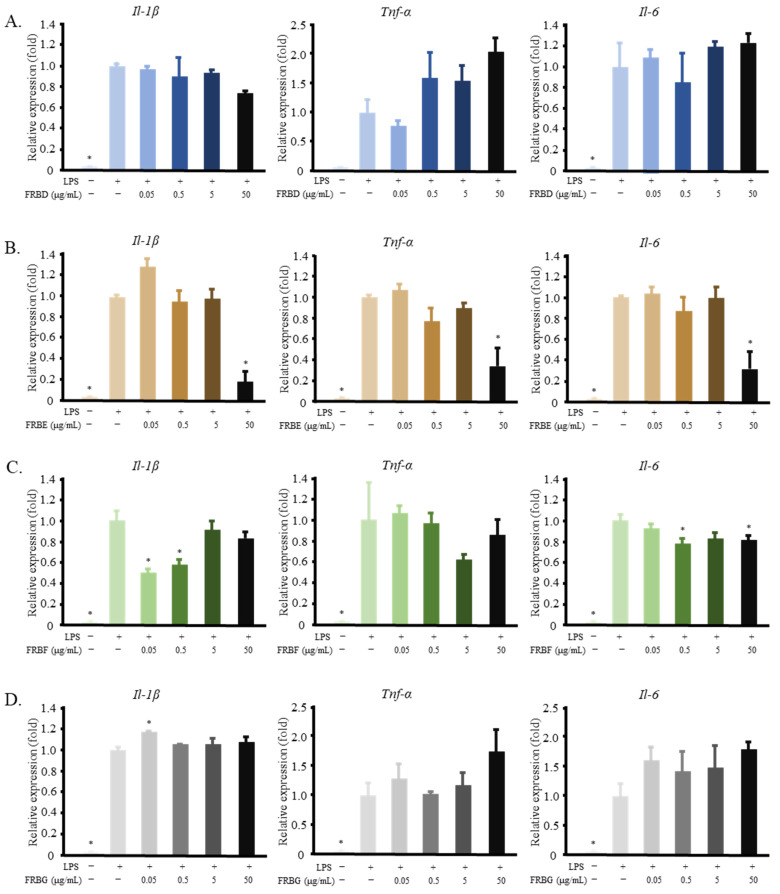
Fractionation of FRBA using a Sep-Pak Plus C18 cartridge resulted in fractions D–G, some of which possess the ability to reduce the inflammatory reaction of LPS-treated RAW 264.7 cells. (**A**) FRBD did not affect the expression of *Il-1β*, *Il-6*, or *Tnf-α*; (**B**) FRBE decreased the mRNA levels of *Il-1β, Il-6*, and *Tnf-α*; (**C**) FRBF reduced the expression of *Il-1β* and *Il-6*; (**D**) The FRBR treatment did not affect the mRNA levels of the pro-inflammatory cytokines in the LPS-activated RAW 264.7 cells. The data are represented as mean ± SE, *n* = 3. Significant differences between the LPS group and the other groups (Dunnet’s analysis, *p* < 0.05) are marked with asterisks (*).

In this fractionation stage, FRBA was further separated into different fractions by solid-phase fractionation using a Sep-Pak C18 cartridge for column chromatography. The fractions produced (FRBD, FRBE, FRBF, and FRBG) were evaluated again for their ability to suppress the LPS-induced pro-inflammatory cytokines in RAW 264.7 cells. FRBD and FRBG did not show any anti-inflammatory effects, as is shown in Figure 3A,D. In contrast, FRBE reduced the mRNA expression of *Il-1β*, *Tnf-α*, and *Il-6* (Figure 3B), and FRBF lowered the mRNA level of *Il-1β* and *Tnf-α* in the LPS-stimulated RAW 264.7 cells (Figure 3C).

### 2.4. Tryptophan Microbial Metabolites in FRB

**Figure 4:**
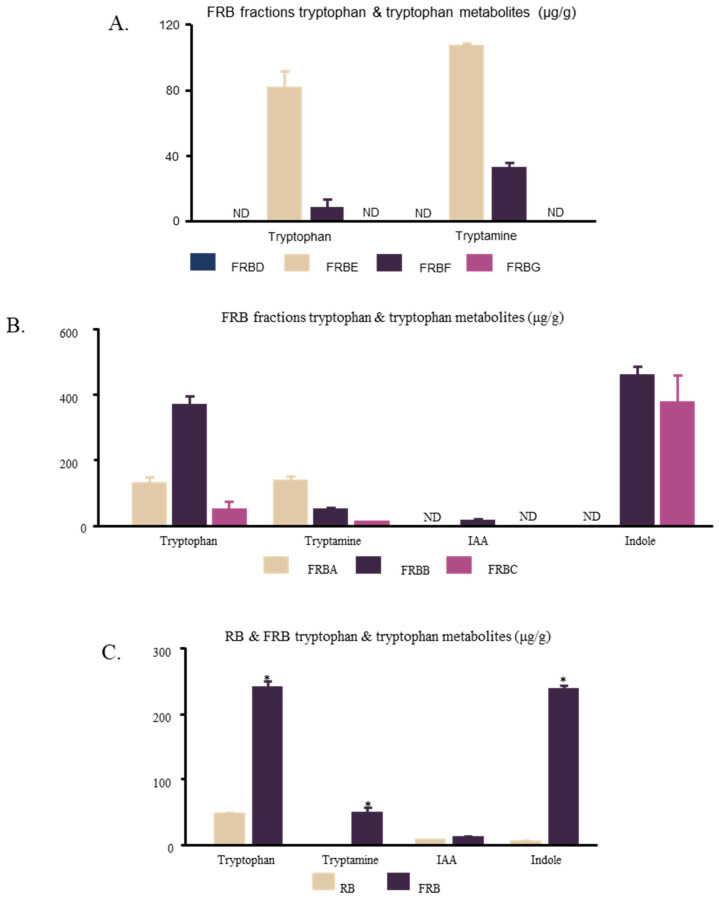
FRB fractions were found to have various levels of tryptophan, tryptamine, indole-3-acetic acid, and indole. (**A**) In the second stage of the FRB fractionation, tryptophan and tryptamine were only found in FRBE and FRBD; (**B**) Solvent extraction of FRB resulted in three different fractions with varying levels of tryptophan and tryptophan metabolites; (**C**) FRB had a significantly higher level of tryptophan, tryptamine, and indole in comparison to RB. The data are represented as mean ± SE, *n* = 3. Significant differences between the groups (*t*-test, *p* < 0.05) are marked with asterisks (*).

Previous studies have indicated that tryptophan and its secondary microbial metabolites, such as tryptamine, indole acetic acid, and indole, play key roles in the ability of FRB to ameliorate dextran sodium sulfate (DSS)-induced colitis [4,27,28]. While this effect was hypothesized to be the result of the ability of the tryptophan metabolites to regulate innate lymphoid cells [4,28,29], tryptophan metabolites in the gut, such as indole acetate and tryptamine, have been suggested to regulate the inflammatory responses of macrophages [30]. Thus, the amounts of tryptophan and tryptamine in FRBD, FRBE, FRBF, and FRBG were evaluated. Tryptophan and tryptamine were not detected in the FRBD or FRBG. In contrast, FRBE and FRBF contained both compounds. FRBE contained significantly higher amounts of both tryptophan and tryptamine (Figure 4A). The amounts of tryptophan and its metabolites were also measured in FRBA, FRBB, and FRBC (Figure 4B). Among these three fractions, FRBA contained the highest amount of tryptamine, whereas FRBB contained the highest amount of tryptophan. Furthermore, a comparison between FRB and RB showed that FRB contained significantly higher amounts of tryptophan, tryptamine, indole-3 acetic acid, and indole, all of which are secondary microbial metabolites of tryptophan (Figure 4C).

### 2.5. FRBE Anti-Inflammatory Capacity Is Dependent on Aryl Hydrocarbon Receptor (AHR) Activity

**Figure 5:**
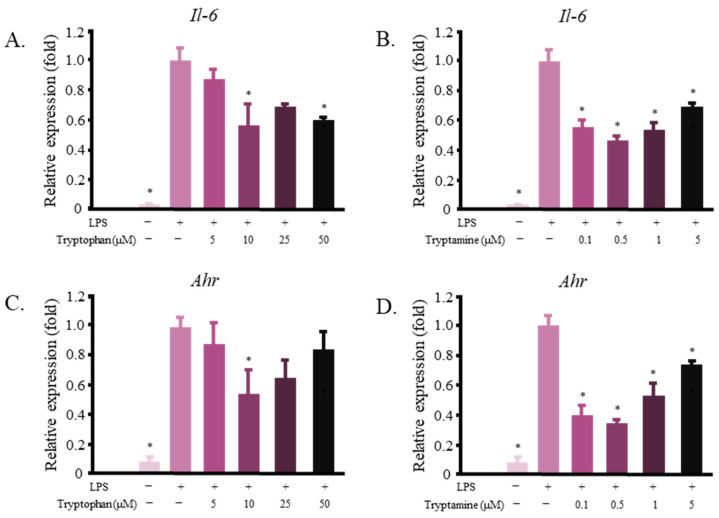
Tryptophan and its microbial metabolites affect the expression of *Il-6* and *Ahr* in LPS-activated RAW 264.7 cells. (**A**) Tryptophan decreased the mRNA level of *Il-6*; (**B**) Tryptamine decreased the expression of *Il-6*; (**C**,**D**) Tryptophan and tryptamine lowered the level of *Ahr*. The data are presented as mean values, with error bars representing the standard errors, *n* = 3. The data were evaluated against the LPS group (Dunnett’s analysis, * *p* < 0.05).

Tryptamine [31] and various other tryptophan metabolites are known as AHR ligands [32]. This receptor is also known to modulate inflammatory reactions in multiple cells in the body [30,33,34,35]. We investigated whether tryptophan and tryptamine could decrease the levels of pro-inflammatory markers in RAW 264.7 cells. Figure 5A,B shows that both tryptophan and tryptamine decreased the mRNA expression level of *Il-6* in LPS-stimulated RAW 264.7 cells. Tryptophan and tryptamine treatments also affected the *Ahr* mRNA levels in the same cells (Figure 5C,D). Tryptamine affected the *Il-6* mRNA production at a lower dose than tryptophan, suggesting that this compound may play a larger role in FRB’s inflammation-reducing activity.

**Figure 6:**
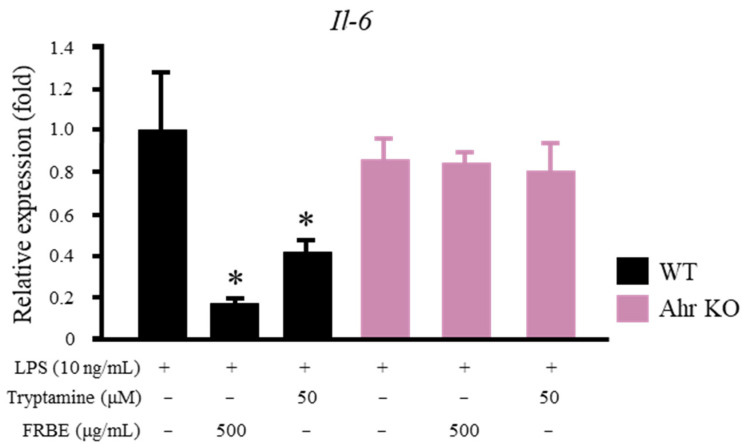
Effect of the FRBE and tryptamine treatments on LPS-treated mouse intraperitoneal macrophages. FRBE and tryptamine reduced the mRNA levels of *Il-6* in the intraperitoneal macrophages of wild-type (WT) mice but not in *Ahr*^−/−^ (*Ahr* Knockout) intraperitoneal macrophages. The data are presented as mean values, with error bars representing the standard errors, *n* = 3. The data were evaluated against the LPS group (Two way ANOVA, Dunnett’s analysis, * *p* < 0.05).

Whether the pro-inflammatory cytokine-reducing effects of FRBE and tryptamine were achieved via AHR activity needs to be confirmed. Macrophages were isolated from the intraperitoneal cavity of *Ahr*-deficient and wild-type mice. The cells were then treated with tryptamine or FRBE before the LPS stimulation and analyzed. Both the FRBE and tryptamine treatments were able to lower the *Il-6* mRNA level in the intraperitoneal macrophages of the wild-type mice (Figure 6). In the *Ahr*-deficient macrophages, however, neither of these substances affected the expression of *Il-6* (Figure 6).

## 3. Discussion

Rice bran contains various bioactive compounds that have been shown to possess anti-inflammatory properties. For instance, feruloyl esters of triterpene alcohols [36], γ-oryzanol [37,38], tocotrienols, tocopherols [38,39], geranylgeraniol [40,41,42], and phenolic compounds from rice bran [43] were found to repress inflammatory responses in various models. Fermented rice bran was used in this study. The fermentation process has been suggested to improve the sensory aspect of rice bran [44], produce new bioactive compounds that may contribute to its inflammation-modulating effect [45,46], and enhance the bioavailability of bioactive compounds in rice bran. The solvent extraction of FRB produced a higher yield than the RB solvent extraction. This result supports our hypothesis that fermentation increases the accessibility of nutrients and functional ingredients in rice bran. Figure 1 shows the differences in the RB and FRB treatments in regulating the inflammatory response of macrophages to LPS. Both treatments exhibited anti-inflammatory effects. However, the FRB treatment lowered the expression of *Il-6*, whereas the RB treatment did not. This result is in line with previous studies [3,4,27], which found that FRB supplementation was more effective in mitigating DSS-induced inflammation in mouse intestines than RB supplementation. Interestingly, high concentrations of FRB seemingly increased the mRNA level of *Il-6* (Figure 1C). Despite containing a vast array of anti-inflammatory compounds, immunostimulatory compounds have also been identified in rice bran [47,48]. A glycoprotein fraction was reported to increase the level of various cytokines, such as TNF-α, IL-1β, IL-6, and IL-10, in RAW 264.7 cells [47].

Solvent extraction is a common method for the isolation of bioactive compounds from rice products. Water and ethanol are typical polar solvents used to isolate rice bioactive compounds, whereas *n*-hexane is a common non-polar solvent for isolating bioactive compounds from rice bran. Hot water can be used to extract phenols, γ-oryzanol, and the vitamins B1, B2, and B3 [14,49]. Additionally, the water extracts of rice bran can be treated to produce glycans, glycoproteins [47], arabinoxylan, and feruloylated oligosaccharides [45,46,50], all of which have immunomodulatory effects. Ethanol can be used to extract phenols, γ-oryzanol, vitamin E, and ferulic acid [14,38,49,51], whilst *n*-hexane can dissolve γ-oryzanol and vitamin E [14]. In this study, defatted rice bran was used to produce FRB. This might explain the low yield of the FRB *n*-hexane fraction (FRBC, 4.99 ± 0.661%). The yield of the hot water extract (FRBA, 31.5 ± 0.062%) was higher than that of the 50% ethanol extract (FRBB, 25.1 ± 1.60%). Figure 3 shows that both FRBA and FRBB have anti-inflammatory effects. Considering that there is some overlap between the bioactive compounds that can be extracted from the hot water extract and the ethanol extract, it is likely that the same anti-inflammatory compound is present in both the FRBA and FRBB fractions. Further separation of the FRBA produced the FRBE and FRBF fractions. These two fractions also showed anti-inflammatory activities (Figure 4B,C). However, FRBF also contains immunomodulatory compounds that appear at higher doses.

The fermentation process was observed to change the composition of rice bran. FRB has a lower content of carbohydrates in comparison to rice bran. However, FRB has a higher content of protein, lipid, ash, total phenolic, and dietary fiber [26]. The dietary supplementation of FRB increases the levels of short-chain fatty acids (SCFAs) in the stool. SCFAs are known to be able to induce T-cell apoptosis, thereby preventing the development of severe colitis. Thus, the increasing level of dietary fiber after rice bran fermentation might prevent the development of colitis in this animal model [4]. The FRB supplementation also increased the levels of tryptophan and tryptamine in feces and serum. Tryptophan and tryptamine were hypothesized to act as AHR ligands and, consequently, to regulate the differentiation of Th17 cells, regulatory T cells, or Th22 cells. The regulation of these immune cells is thought to promote intestinal barrier function and intestinal homeostasis [4]. Tryptophan metabolites were also suggested to be able to regulate IL-22 production by innate lymphoid cells [28], although gut microbiota-produced indole acetate and tryptamine have been shown to ameliorate inflammatory responses in hepatocytes and macrophages [30].

Bacterial metabolism converts tryptophan to indole, indole-3-acetic acid, and tryptamine [32]. These compounds were found in significantly higher amounts in FRB than in RB (Figure 5A). They were also found in most FRB fractions, namely, FRBA, FRBB, FRBC, FRBE, and FRBF (Figure 5B,C). All of these FRB fractions have been shown to suppress inflammation. In their pure form, these indole compounds affected the mRNA level of *Il-6* in murine macrophages (Figure 6). Although both tryptophan and tryptamine were found in the FRBE fraction, a lower concentration of tryptamine was needed to alleviate the increase in the level of the pro-inflammatory cytokine *Il-6*. Tryptamine probably plays a larger role in suppressing inflammation in RAW 264.7 cells. It was discovered that while FRBE reduced the *Il-6* level in RAW 264.7 cells at a concentration of 50 μg/mL, this fraction only contains 0.03 μM tryptamine. Figure 5B shows that the concentration of tryptamine needed to decrease the level of *Il-6* was 0.1 μM. Together, these results suggest that tryptamine, tryptophan, and possibly other indole compounds in FRB synergistically cooperate to modulate the inflammatory reaction of LPS-stimulated macrophages.

Indole, indole-3-acetic acid, and tryptamine have been established as AHR ligands with various degrees of affinities [52]. AHR was reported to have the ability to modulate not only the drug/xenobiotic metabolism but also immune system responses [35,53,54]. AHR forms a complex with Stat1 and NF-κB in LPS-activated macrophages. This affects the regulation of the *Il-6* promoter activity [55]. This study potentially explains how tryptophan and tryptamine treatments decrease the level of *Il-6* (Figure 6). Moreover, FRB contains not only tryptamine and tryptophan but also other AHR ligands, such as indole and IAA (Figure 4C). Although AHR activation has been shown to reduce inflammation [55,56], other studies have also reported that AHR activation is responsible for inducing IL-6 [57]. Different ligands have been suggested to produce a specific structure of AHR and alter its DNA binding activity. Consequently, gene induction by AHR is thought to be ligand-specific [58]. Figure 6 also shows that different concentrations of tryptamine and tryptophan elicit different responses from macrophages. AHR activity and its gene-inducing effect may also be affected by ligand availability. The presence of various AHR ligands in FRB in different concentrations might have contributed to the increased level of *Il-6* mRNA due to FRB treatment in high concentrations (Figure 1B). While this study has shown that the application of both tryptophan and tryptamine affects *Ahr* expression and regulates the macrophage inflammatory response, its correlation and mechanism need to be further explored.

The succession of microbiota that was used in this study might also produce bioactive compounds other than tryptophan and its metabolites. For example, *Aspergillus kawachii* fermentation has been reported to increase the anti-oxidative properties of yellow onion [59] and rice koji [60], along with elevating the anti-cancer activity of silkworm larvae [61]. *Aspegillus kawachii* has also been shown to produce glucosylceramides in rice koji [62]. Glucosylceramides have shown an anti-inflammatory capacity on LPS-stimulated RAW 264.7 cells [63]. However, it is important to note that glucosylceramides did not affect IL-6 production in this system [63]. Lactic acid bacteria are known to act as probiotics. They have also been documented to produce some bioactive compounds. Additionally, γ-Aminobutyric acid can be produced by *Levilactobacillus brevis* [64]. This compound has strong immunoregulatory effects on mesenteric lymph nodes [65]. Another species of lactic acid bacteria, *Lacticaseibacillus rhamnosus*, produced some effector molecules that can prevent LPS-induced intestinal injuries by modulating the production of TNF-α [66]. It is possible that these bioactive compounds produced by this combination of microbiota also contributed to the FRB’s inflammation suppressing effect.

## 4. Materials and Methods

### 4.1. Materials

The defatted rice bran was fermented with *Aspergillus kawachii*, followed by a mixture of lactic acid bacteria (FRB). The non-fermented rice bran (RB) was prepared using the same procedure without the addition of either microbiota culture. A detailed explanation of the FRB preparation has been provided by Islam et al. [4] and Alauddin et al. [19]. RB and FRB were supplied by the Sunstar Company (Tokyo, Japan). The ʟ-tryptophan, 5-OH tryptophan, tryptamine, indole-3-acetic acid, and indole were purchased from Sigma-Aldrich (St. Louis, MO, USA).

### 4.2. The First Stage of Extraction and Fractionation of Compounds in FRB

Three different solvents were used in the first stage of the extraction process. The first solvent comprised 60 mL of hot water (60 °C), the second was 50 mL of 50% ethanol, and the last one was 50 mL of *n*-hexane solution. The FRB (10 g, dry weight) was then mixed with each solvent overnight with constant agitation. Subsequently, the mixtures were filtered with Advantec Filter Paper (Toyo Roshi Kaisha Ltd., Tokyo, Japan). A total of 60 mL of water was used instead of 50 mL to counter the amount of water that evaporated from the overnight mixture. This extra amount of liquid also ensures ease in the filtration process. The hot water extract was then lyophilized for 72 h. The dried extract will, henceforth, be referred to as FRBA (yield 31.5 ± 0.062%). The 50% ethanol extract was evaporated by vacuum rotary evaporation (Spin Dryer Lite VC-36R, TAITEC Corporation, Saitama, Japan) for 72 h at 50 °C. The product was referred to as FRBB (yield 25.1 ± 1.60%). The *n*-hexane extract was also evaporated by vacuum rotary evaporation for 2 h at 50 °C. This *n*-hexane fraction will be denoted as FRBC throughout the manuscript (yield 4.99 ± 0.661%). For comparison, a similar process was applied to the RB. In general, the fractionation of FRB tends to result in a higher yield than RB (the yield of RBA was 9.85 ± 0.628%; the yield of RBB was 6.62 ± 0.083%; the yield of RBC 3.99 ± 0.631%).

### 4.3. The Second Stage of Fractionation and Isolation of Active Compounds in FRB

**Figure 7:**
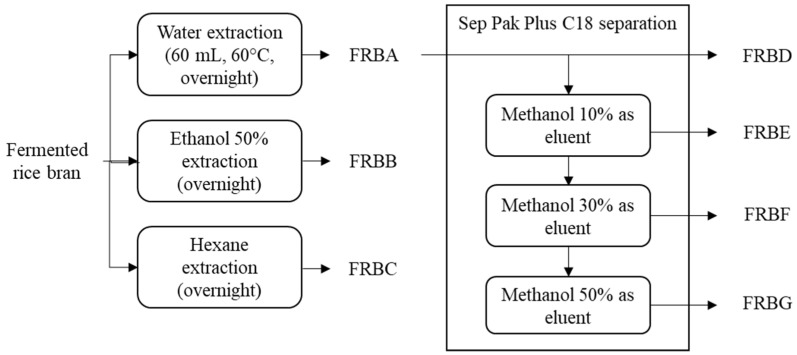
Scheme of the current experimental design.

Only the hot-water fraction (FRBA) was used in the second fractionation stage. Sep-Pak^®^ Plus C18 cartridges (Waters Corporation, Milford, MA, USA) were used in this stage. The Sep-Pak Plus C18 cartridge was activated using 2 mL of methanol before use, followed by 2 mL of dH_2_O. The FRBA (0.25 g) was diluted in 5 mL of dH_2_O and, subsequently, loaded into a syringe. The syringe was then attached to a Sep-Pak Plus C18 cartridge and the FRBA solution was flushed out through the cartridge. The solution that was filtered from this stage will be referred to as FRBD (yield 52.4 ± 0.306%). The syringe was then filled with 2 mL of dH_2_O to wash the Sep-Pak Plus C18 cartridge and the FRB compounds trapped inside it. This step was followed by an extraction using 10%, 30%, and 50% of methanol as the eluents. The FRB fractions from these extracts will, henceforth, be referred to as FRBE (yield 1.83 ± 0.196%), FRBF (yield 4.08 ± 0.343%), and FRBG (yield 1.77 ± 0.078%). The complete extraction and isolation processes are shown in Figure 7.

### 4.4. Maintenance and Treatment of RAW 264.7 Cells

The RAW 264.7 cell culture was provided by the RIKEN BioResource Research Center (Tsukuba, Japan). This cell line is a murine macrophage cell line obtained from a male mouse induced with the Abelson murine leukemia virus. This cell line is used in obtaining Figure 1, Figure 2, Figure 3 and Figure 5 in this manuscript. Eagle’s minimum essential medium (EMEM; Sigma Aldrich, Tokyo, Japan) supplemented with 10% fetal bovine serum (Biosera, East Sussex, UK), 100 μg/mL streptomycin, 100 U/mL penicillin (Gibco, Thermo Fisher Scientific, Tokyo, Japan), MEM non-essential amino acids (Sigma Aldrich, Hampshire, UK), and 10 mM Na-pyruvate (Gibco) was used for the cell maintenance and treatment. The cells were maintained at 37 °C in a humidified 5% CO_2_ atmosphere. To evaluate the anti-inflammatory effects of the RB, FRB, FRB fractions, tryptophan, and tryptamine, the cells were seeded in 6 cm dishes (1.5 × 10^6^ cells/dish). After an overnight incubation, the cell medium was replaced with another medium containing various concentrations of the treatment agent, and the dishes were returned to the incubator. Twenty-four hours after the medium was changed, the cells were stimulated with LPS (1 μg/mL, *Escherichia coli* serotype O111: B4, L2630: Sigma-Aldrich) for 3 h before harvesting.

### 4.5. High-Performance Liquid Chromatography (HPLC) Analysis for Tryptophan and Its Microbial Metabolites

Tryptophan and its metabolite contents in FRB were analyzed using fluorescence HPLC. The analysis was conducted using a previously described method [67] with modifications. In short, the HPLC analysis was performed with an Atlantis T3 column (4.6 × 50 mm, 5 μm; Waters, Milford, MA, USA) at 30 °C and at a flow rate of 1.0 mL/min. The separation was conducted using a gradient of 10 mM of HCOONH_4_ (pH 3.4), acetonitrile, and distilled water. The acetonitrile and distilled water (HPLC grade) were supplied by Kanto Chemical Co., Inc. (Tokyo, Japan). The total HPLC run time was 60 min for each sample, and 20 μL of the sample was injected into each cycle. The fluorescence was detected at 300 nm excitation and 355 nm emission.

### 4.6. Quantitative Reverse Transcriptase Mediated Polymerase Chain Reaction (qRT-PCR)

A qRT-PCR analysis was performed, as described by Agista et al. [21]. Briefly, the cells were harvested after a treatment with ISOGEN (Nippon Gene Co., Ltd., Tokyo, Japan), and the RNA from the cell samples was isolated and purified. The purified RNA was used as a template to produce cDNA. The cDNA produced was analyzed by qRT-PCR using a Bio-Rad CFX Connect (Hercules, CA, USA). The mRNA level of a housekeeping gene, the eukaryotic elongation factor 1-alpha 1 (*Eef1a1*), was used to normalize the mRNA expression levels of other genes. Table 1 lists the gene-specific primers used in this study.

### 4.7. Isolation of Mouse Intraperitoneal Macrophages

The intraperitoneal macrophages were induced in both the wild-type and *Ahr*−/− mice (C57BL/6N) by intraperitoneal injections of fluid thioglycollate media (Nissui Pharmaceutical Co., Ltd., Tokyo, Japan). Thioglycollate-induced peritonitis is a method often used to collect macrophages from the intraperitoneal cavity [55,68]. The collected cells were used in obtaining Figure 6. After 48–72 h, the mice were euthanized by cervical dislocation and soaked in ethanol. The intraperitoneal macrophages were then harvested by injecting a CMF-Hanks solution (4.0 g of NaCl, 0.024 g of Na_2_HPO_4_, 0.03 g of KH_2_PO_4_, and 0.5 g of glucose in 500 mL of purified water) into the mice’s intraperitoneal cave. The macrophages were centrifuged and washed with the CMF-Hanks solution. The red blood cells in the cell pellets were lysed with an NH_4_Cl solution, and the cells were washed once again. The mixture was then centrifuged and the aqueous solution was removed. The remaining cell pellets were mixed with an RPMI 1640 medium (Sigma-Aldrich). The cells were plated in 10 cm dishes at a concentration of 1.0 × 10^6^ cells/mL before incubation for 3 h. After the incubation period, the cells were collected and re-plated into 6-cm dishes. The cells were incubated overnight, and then their medium was changed to a medium containing either DMSO, FRBE (a fraction of FRB), or tryptamine. The cells were returned to the incubator for another 24 h before 10 ng/mL of LPS was added to each plate. After the addition of LPS, the cells were incubated for another 3 h. The RNA from the cells was isolated and purified for the qRT-PCR analysis.

### 4.8. Statistical Analysis

The results of this study are presented as mean ± standard error (SE). The SigmaPlot version 12.5 (San Jose, CA, USA) was used to perform the statistical analysis. The data were evaluated using a one-way analysis of variance followed by a Dunnett’s test or a Tukey–Kramer post-hoc analysis. The statistical differences between the groups are denoted by the levels indicated in each figure.

## 5. Conclusions

The dual fermentation of rice bran with *Aspergillus kawachii* and a mixture of lactic acid bacteria is suspected to change the nutritional content and accessibility of rice bran. FRB was found to have a higher content of tryptophan and its bacterial metabolites, tryptamine and indoles. Despite these differences, both RB and FRB were observed to be able to reduce the expression of pro-inflammatory cytokines, such as *Il-1β* and *iNos*, in LPS-activated murine macrophages. Both were also able to increase the level of the anti-inflammatory cytokine *Il-10*. Interestingly, only the FRB treatment lowered the level of *Il-6*. The fractionation of FRB indicated that tryptamine plays an important role in the anti-inflammatory effect of FRB. Tryptophan and tryptamine reduced the expression of *Il-6* in LPS-activated RAW 264.7 cells. Tryptophan and its microbial metabolites have also been found to alter the expression of *Ahr*, which is known to modulate the inflammatory response in macrophages. Nonetheless, the mechanism and correlation between the AHR activation and the tryptophan metabolites in FRB that can mitigate the inflammatory response in macrophages remain to be investigated.

## Figures and Tables

**Table 1:** List of genes and their primer sequences.

Gene Name	Forward Primer	Reverse Primer	Accession Number
Eukaryotic translation elongation factor 1 alpha 1 (*Eef1a1*)	GATGGCCCCAAATTCTTGAAG	GGACCATGTCAACAATGGCAG	NM_010106.2
Interleukin-1β (*Il-1β*)	CTGTGTCTTTCCCGTGGACC	CAGCTCATATGGGTCCGACA	NM_008361.4
Tumor necrosis factor α (*Tnf-α*)	GACGTGGAACTGGCAGAAGAG	TCTGGAAGCCCCCCATCT	NM_013693.3, NM_001278601.1
Interleukin-6 (*Il-6*)	AGAGGAGACTTCACAGAGGATACCA	AATCAGAATTGCCATTGCACAAC	NM_031168.2, NM_001314054.1
Inducible nitric oxide synthase (*iNos*)	CAGGTGCACACAGGCTACT	GAGCACGCTGAGTACCTCATT	NM_001313922.1, NM_001313921.1, NM_010927.4
Interleukin 10 (*Il-10*)	TGAATTCCCTGGGTGAGAAGCTGA	TGGCCTTGTAGACACCTTGGTCTT	NM_010548.2

## Data Availability

Not applicable.

## References

[1] Josefs T., Barrett T.J., Brown E.J., Quezada A., Wu X., Voisin M., Amengual J., Fisher E.A. (2020). Neutrophil extracellular traps promote macrophage inflammation and impair atherosclerosis resolution in diabetic mice. JCI Insight.

[2] van Diepen J.A., Robben J.H., Hooiveld G.J., Carmone C., Alsady M., Boutens L., Bekkenkamp-Grovenstein M., Hijmans A., Engelke U.F.H., Wevers R.A. (2017). SUCNR1-mediated chemotaxis of macrophages aggravates obesity-induced inflammation and diabetes. Diabetologia.

[3] Rusbana T.B., Agista A.Z., Saputra W.D., Ohsaki Y., Watanabe K., Ardiansyah A., Budijanto S., Koseki T., Aso H., Komai M. (2020). Supplementation with Fermented Rice Bran Attenuates Muscle Atrophy in a Diabetic Rat Model. Nutrients.

[4] Islam J., Koseki T., Watanabe K., Ardiansyah, Budijanto S., Oikawa A., Alauddin M., Goto T., Aso H., Komai M. (2017). Dietary supplementation of fermented rice bran effectively alleviates dextran sodium sulfate-induced colitis in mice. Nutrients.

[5] Scrivo R., Vasile M., Bartosiewicz I., Valesini G. (2011). Inflammation as “common soil” of the multifactorial diseases. Autoimmun. Rev..

[6] Hartupee J., Mann D.L. (2016). Role of inflammatory cells in fibroblast activation. J. Mol. Cell. Cardiol..

[7] Frangogiannis N.G. (2018). Cell biological mechanisms in regulation of the post-infarction inflammatory response. Curr. Opin. Physiol..

[8] Maslowski K.M., Vieira A.T., Ng A., Kranich J., Sierro F., Yu D., Schilter H.C., Rolph M.S., Mackay F., Artis D. (2009). Regulation of inflammatory responses by gut microbiota and chemoattractant receptor GPR43. Nature.

[9] Tomita K., Freeman B.L., Bronk S.F., Lebrasseur N.K., White T.A., Hirsova P., Ibrahim S.H. (2016). CXCL10-Mediates Macrophage, but not Other Innate Immune Cells-Associated Inflammation in Murine Nonalcoholic Steatohepatitis. Sci. Rep..

[10] Wang C., Petriello M.C., Zhu B., Hennig B. (2019). PCB 126 induces monocyte/macrophage polarization and inflammation through AhR and NF-κB pathways. Toxicol. Appl. Pharmacol..

[11] Luo D., Guo Y., Cheng Y., Zhao J., Wang Y., Rong J. (2017). Natural product celastrol suppressed macrophage M1 polarization against inflammation in diet-induced obese mice via regulating Nrf2/HO-1, MAP kinase and NF-κB pathways. Aging.

[12] Hefnawy H.T.M., El-shourbagy G. (2014). A Chemical Analysis and Antioxidant Activity of Polysaccharide Extracted from Rice Bran. World J. Dairy Food Sci..

[13] Dapar M.L.G., Garzon J.F., Demayo C.G. (2013). Cytotoxic activity and Antioxidant Potentials of hexane and Methanol extracts of IR64 Rice bran against Human Lung (A549) and Colon (HCT116) Carcinomas. Int. Res. J. Biol. Sci..

[14] Itharat A., Uttama S., Makchuchit S. (2016). Anti-Inflammatory Activities of Constituents in Sang Yod Rice Extracts, γ-Oryzanol, Vitamins E, B1, B2 and B3, Using Inhibitory Effects on Nitric Oxide (NO) Production in Lipopolysaccharide (LPS) Activated RAW 264.7 Murine Macrophage Cells. Med. Aromat. Plants.

[15] Justo M.L., Candiracci M., Dantas A.P., de Sotomayor M.A., Parrado J., Vila E., Herrera M.D., Rodriguez-Rodriguez R. (2013). Rice bran enzymatic extract restores endothelial function and vascular contractility in obese rats by reducing vascular inflammation and oxidative stress. J. Nutr. Biochem..

[16] Nagendra Prasad M.N., Sanjay K.R., Shravy Khatokar M., Vismaya M.N., Najunda Swamy S. (2011). Health Benefits of Rice Bran —A Review. J. Nutr. Food Sci..

[17] de Castro Oliveira M.G., Bassinello P.Z., da Silva Lobo V.L., Rinaldi M.M. (2012). Stability and microbiological quality of rice bran subjected to different heat treatments. Food Sci. Technol..

[18] Satter M.A., Ara H., Jabin S.A., Abedin N., Azad A.K., Hossain A., Ara U. (2014). Nutritional Composition and Stabilization of Local Variety Rice Bran BRRI-28. Int. J. Sci. Technol..

[19] Kum S.-J., Yang S.-O., Lee S.M., Chang P.-S., Choi Y.H., Lee J.J., Hurh B.S., Kim Y.-S. (2015). Effects of aspergillus species inoculation and their enzymatic activities on the formation of volatile components in fermented soybean paste (doenjang). J. Agric. Food Chem..

[20] Futagami T., Mori K., Wada S., Ida H., Kajiwara Y., Takashita H., Tashiro K., Yamada O., Omori T., Kuhara S. (2015). Transcriptomic analysis of temperature responses of *Aspergillus kawachii* during barley koji production. Appl. Environ. Microbiol..

[21] Ramos C.L., Thorsen L., Schwan R.F., Jespersen L. (2013). Strain-specific probiotics properties of *Lactobacillus fermentum*, *Lactobacillus plantarum* and *Lactobacillus brevis* isolates from Brazilian food products. Food Microbiol..

[22] Erkkilä S., Suihko M.-L., Eerola S., Petäjä E., Mattila-Sandholm T. (2001). Dry sausage fermented by *Lactobacillus rhamnosus* strains. Int. J. Food Microbiol..

[23] Moslehishad M., Ehsani M.R., Salami M., Mirdamadi S., Ezzatpanah H., Naslaji A.N., Moosavi-Movahedi A.A. (2013). The comparative assessment of ACE-inhibitory and antioxidant activities of peptide fractions obtained from fermented camel and bovine milk by *Lactobacillus rhamnosus* PTCC 1637. Int. Dairy J..

[24] Villena J., Chiba E., Tomosada Y., Salva S., Marranzino G., Kitazawa H., Alvarez S. (2012). Orally administered *Lactobacillus rhamnosus* modulates the respiratory immune response triggered by the viral pathogen-associated molecular pattern poly(I:C). BMC Immunol..

[25] Zommiti M., Cambronel M., Maillot O., Barreau M., Sebei K., Feuilloley M., Ferchichi M., Connil N. (2018). Evaluation of probiotic properties and safety of *Enterococcus faecium* isolated from artisanal Tunisian Meat “Dried Ossban”. Front. Microbiol..

[26] Alauddin M., Shirakawa H., Koseki T., Kijima N., Ardiansyah N., Budijanto S., Islam J., Goto T., Komai M. (2016). Fermented rice bran supplementation mitigates metabolic syndrome in stroke-prone spontaneously hypertensive rats. BMC Complement. Altern. Med..

[27] Agista A., Rusbana T., Islam J., Ohsaki Y., Sultana H., Hirakawa R., Watanabe K., Nochi T., Ardiansyah, Budijanto S. (2021). Fermented rice bran supplementation prevents the development of intestinal fibrosis due to DSS-induced inflammation in mice. Nutrients.

[28] Islam J., Agista A.Z., Watanabe K., Nochi T., Aso H., Ohsaki Y., Koseki T., Komai M., Shirakawa H. (2022). Fermented rice bran supplementation attenuates chronic colitis-associated extraintestinal manifestations in female C57BL/6N mice. J. Nutr. Biochem..

[29] Islam J., Sato S., Watanabe K., Watanabe T., Ardiansyah, Hirahara K., Aoyama Y., Tomita S., Aso H., Komai M. (2017). Dietary tryptophan alleviates dextran sodium sulfate-induced colitis through aryl hydrocarbon receptor in mice. J. Nutr. Biochem..

[30] Krishnan S., Ding Y., Saedi N., Choi M., Sridharan G.V., Sherr D.H., Yarmush M.L., Alaniz R.C., Jayaraman A., Lee K. (2018). Gut Microbiota-Derived Tryptophan Metabolites Modulate Inflammatory Response in Hepatocytes and Macrophages. Cell Rep..

[31] Bergander L.V., Cai W., Klocke B., Seifert M., Pongratz I. (2012). Tryptamine serves as a proligand of the AhR transcriptional pathway whose activation is dependent of monoamine oxidases. Mol. Endocrinol..

[32] Hubbard T.D., Murray I.A., Perdew G.H. (2015). Special section on drug metabolism and the microbiome—Minireview indole and tryptophan metabolism: Endogenous and dietary routes to ah receptor activation. Drug Metab. Dispos..

[33] Goettel J.A., Gandhi R., Kenison J.E., Yeste A., Murugaiyan G., Sambanthamoorthy S., Griffith A.E., Patel B., Shouval D.S., Weiner H.L. (2016). AHR Activation Is Protective against Colitis Driven by T Cells in Humanized Mice. Cell Rep..

[34] Qiu J., Guo X., Chen Z.-M.E., He L., Sonnenberg G.F., Artis D., Fu Y.-X., Zhou L. (2013). Group 3 innate lymphoid cells inhibit T-cell-mediated intestinal inflammation through aryl hydrocarbon receptor signaling and regulation of microflora. Immunity.

[35] Zhu J., Luo L., Tian L., Yin S., Ma X., Cheng S., Tang W., Yu J., Ma W., Zhou X. (2018). Aryl hydrocarbon receptor promotes IL-10 expression in inflammatory macrophages through Src-STAT3 signaling pathway. Front. Immunol..

[36] Akihisa T., Yasukawa K., Yamaura M., Ukiya M., Kimura Y., Shimizu N., Arai K. (2000). Triterpene alcohol and sterol ferulates from rice bran and their anti- inflammatory effects. J. Agric. Food Chem..

[37] Rao Y.P.C., Sugasini D., Lokesh B. (2016). Dietary gamma oryzanol plays a significant role in the anti-inflammatory activity of rice bran oil by decreasing pro-inflammatory mediators secreted by peritoneal macrophages of rats. Biochem. Biophys. Res. Commun..

[38] Bhatia H.S., Baron J., Hagl S., Eckert G.P., Fiebich B.L. (2016). Rice bran derivatives alleviate microglia activation: Possible involvement of MAPK pathway. J. Neuroinflamm..

[39] Shen J., Yang T., Xu Y., Luo Y., Zhong X., Shi L., Hu T., Guo T., Nie Y., Luo F. (2018). δ-Tocotrienol, Isolated from Rice Bran, Exerts an Anti-Inflammatory Effect via MAPKs and PPARs Signaling Pathways in Lipopolysaccharide-Stimulated Macrophages. Int. J. Mol. Sci..

[40] Giriwono P.E., Shirakawa H., Ohsaki Y., Hata S., Kuriyama H., Sato S., Goto T., Komai M. (2013). Dietary supplementation of geranylgeraniol suppresses lipopolysaccharide-induced inflammation via the inhibition of NF-κB activation in rats. Eur. J. Nutr..

[41] Giriwono P.E., Shirakawa H., Ohsaki Y., Sato S., Aoyama Y., Ho H.-J., Goto T., Komai M. (2019). Geranylgeraniol Suppresses the expression of IRAK1 and TRAF6 to Inhibit NFκB Activation in Lipopolysaccharide-Induced Inflammatory Responses in Human Macrophage-Like Cells. Int. J. Mol. Sci..

[42] Saputra W.D., Shono H., Ohsaki Y., Sultana H., Komai M., Shirakawa H. (2021). Geranylgeraniol Inhibits Lipopolysaccharide-Induced Inflammation in Mouse-Derived MG6 Microglial Cells via NF-κB Signaling Modulation. Int. J. Mol. Sci..

[43] Xiao J., Zhang R., Wu Y., Wu C., Jia X., Dong L., Liu L., Chen Y., Bai Y., Zhang M. (2020). Rice Bran Phenolic Extract Protects against Alcoholic Liver Injury in Mice by Alleviating Intestinal Microbiota Dysbiosis, Barrier Dysfunction, and Liver Inflammation Mediated by the Endotoxin–TLR4–NF-κB Pathway. J. Agric. Food Chem..

[44] Ardiansyah, Nada A., Rahmawati N., Oktriani A., David W., Astuti R., Handoko D., Kusbiantoro B., Budijanto S., Shirakawa H. (2021). Volatile Compounds, Sensory Profile and Phenolic Compounds in Fermented Rice Bran. Plants.

[45] Fang H.-Y., Chen Y.-K., Chen H.-H., Lin S.-Y., Fang Y.-T. (2012). Immunomodulatory effects of feruloylated oligosaccharides from rice bran. Food Chem..

[46] Lin C.C., Chen H.H., Chen Y.K., Chang H.C., Lin P.Y., Pan I.-H., Chen D.-Y., Chen C.-M., Lin S.Y. (2014). Rice bran feruloylated oligosaccharides activate dendritic cells via toll-like receptor 2 and 4 signaling. Molecules.

[47] Park H.-Y., Yu A.-R., Choi I.-W., Hong H.-D., Lee K.-W., Choi H.-D. (2013). Immunostimulatory effects and characterization of a glycoprotein fraction from rice bran. Int. Immunopharmacol..

[48] Park Y.M., Lee H.Y., Shin D.Y., Lee Y.H., Yang Y.J., Lee H.S., Lee J.O., Choi K.S., Kang J.H., Cho Y.H. (2020). Immunostimulatory Activity of Black Rice Bran in Cyclophosphamide-Induced Immunosuppressed Rats. Nat. Prod. Commun..

[49] Rashid N.Y.A., Razak D.L.A., Jamaluddin A., Sharifuddin S.A., Long K. (2015). Bioactive compounds and antioxidant activity of rice bran fermented with lactic acid bacteria. Malays. J. Microbiol..

[50] Forster G.M., Raina K., Kumar A., Kumar S., Agarwal R., Chen M.-H., Bauer J.E., McClung A.M., Ryan E.P. (2013). Rice varietal differences in bioactive bran components for inhibition of colorectal cancer cell growth. Food Chem..

[51] Limtrakul P., Yodkeeree S., Pitchakarn P., Punfa W. (2016). Anti-inflammatory effects of proanthocyanidin-rich red rice extract via suppression of MAPK, AP-1 and NF-κB pathways in Raw 264.7 macrophages. Nutr. Res. Pract..

[52] Cheng Y., Jin U.-H., Allred C.D., Jayaraman A., Chapkin R.S., Safe S. (2015). Special section on drug metabolism and the microbiome: Aryl hydrocarbon receptor activity of tryptophan metabolites in young adult mouse colonocytes. Drug Metab. Dispos..

[53] Lee Y.H., Lin C.H., Hsu P.C., Sun Y.Y., Huang Y.J., Zhuo J.H., Wang C.Y., Gan Y.L., Hung C.C., Kuan C.Y. (2015). Aryl hydrocarbon receptor mediates both proinflammatory and anti-inflammatory effects in lipopolysaccharide-activated microglia. Glia.

[54] Takamura T., Harama D., Matsuoka S., Shimokawa N., Nakamura Y., Okumura K., Ogawa H., Kitamura M., Nakao A. (2010). Activation of the aryl hydrocarbon receptor pathway may ameliorate dextran sodium sulfate-induced colitis in mice. Immunol. Cell Biol..

[55] Kimura A., Naka T., Nakahama T., Chinen I., Masuda K., Nohara K., Fujii-Kuriyama Y., Kishimoto T. (2009). Aryl hydrocarbon receptor in combination with Stat1 regulates LPS-induced inflammatory responses. J. Exp. Med..

[56] Masuda K., Kimura A., Hanieh H., Nguyen N.T., Nakahama T., Chinen I., Otoyo Y., Murotani T., Yamatodani A., Kishimoto T. (2011). Aryl hydrocarbon receptor negatively regulates LPS-induced IL-6 production through suppression of histamine production in macrophages. Int. Immunol..

[57] Adelibieke Y., Yisireyili M., Ng H.-Y., Saito S., Nishijima F., Niwa T. (2014). Indoxyl sulfate induces IL-6 expression in vascular endothelial and smooth muscle cells through OAT3-mediated uptake and activation of AhR/NF-κB pathway. Nephron Exp. Nephrol..

[58] Denison M.S., Faber S.C. (2017). And now for something completely different: Diversity in ligand-dependent activation of Ah receptor responses. Curr. Opin. Toxicol..

[59] Yang E.-J., Kim S.-I., Park S.-Y., Bang H.-Y., Jeong J.H., So J.-H., Rhee I.-K., Song K.-S. (2012). Fermentation enhances the in vitro antioxidative effect of onion (*Allium cepa*) via an increase in quercetin content. Food Chem. Toxicol..

[60] Miyake Y., Ito C., Itoigawa M., Osawa T. (2007). Isolation of the Antioxidant Pyranonigrin-A from Rice Mold Starters Used in the Manufacturing Process of Fermented Foods. Biosci. Biotechnol. Biochem..

[61] Cho H.-D., Min H.-J., Won Y.-S., Ahn H.-Y., Cho Y.-S., Seo K.-I. (2019). Solid state fermentation process with *Aspergillus kawachii* enhances the cancer-suppressive potential of silkworm larva in hepatocellular carcinoma cells. BMC Complement. Altern. Med..

[62] Hirata M., Tsuge K., Jayakody L.N., Urano Y., Sawada K., Inaba S., Nagao K., Kitagaki H. (2012). Structural determination of glucosylceramides in the distillation remnants of shochu, the japanese traditional liquor, and its production by *Aspergillus kawachii*. J. Agric. Food Chem..

[63] Yeom M., Park J., Lim C., Sur B., Lee B., Han J.-J., Choi H.-D., Lee H., Hahm D.-H. (2015). Glucosylceramide attenuates the inflammatory mediator expression in lipopolysaccharide-stimulated RAW264.7 cells. Nutr. Res..

[64] Lim H.S., Cha I.-T., Roh S.W., Shin H.-H., Seo M.-J. (2017). Enhanced production of gamma-aminobutyric acid by optimizing culture conditions of *Lactobacillus brevis* HYE1 isolated from kimchi, a Korean fermented food. J. Microbiol. Biotechnol..

[65] Bajić S.S., Đokić J., Dinić M., Tomić S., Popović N., Brdarić E., Golić N., Tolinački M. (2020). GABA potentiate the immunoregulatory effects of *Lactobacillus brevis* BGZLS10-17 via ATG5-dependent autophagy in vitro. Sci. Rep..

[66] Gao J., Li Y., Wan Y., Hu T., Liu L., Yang S., Gong Z., Zeng Q., Wei Y., Yang W. (2019). A novel postbiotic from *Lactobacillus rhamnosus* GG with a beneficial effect on intestinal barrier function. Front. Microbiol..

[67] Islam J., Shirakawa H., Nguyen T.K., Aso H., Komai M. (2016). Simultaneous analysis of serotonin, tryptophan and tryptamine levels in common fresh fruits and vegetables in Japan using fluorescence HPLC. Food Biosci..

[68] Geng T., Yan Y., Xu L., Cao M., Xu Y., Pu J., Yan J.C. (2020). CD137 signaling induces macrophage M2 polarization in atherosclerosis through STAT6/PPARδ pathway. Cell. Signal..

